# Opportunities in optical and electrical single-cell technologies to study microbial ecosystems

**DOI:** 10.3389/fmicb.2023.1233705

**Published:** 2023-08-25

**Authors:** Fabian Mermans, Valérie Mattelin, Ruben Van den Eeckhoudt, Cristina García-Timermans, Josefien Van Landuyt, Yuting Guo, Irene Taurino, Filip Tavernier, Michael Kraft, Hira Khan, Nico Boon

**Affiliations:** ^1^Center for Microbial Ecology and Technology (CMET), Department of Biotechnology, Ghent University, Ghent, Belgium; ^2^Department of Oral Health Sciences, KU Leuven, Leuven, Belgium; ^3^Micro- and Nanosystems (MNS), Department of Electrical Engineering (ESAT), KU Leuven, Leuven, Belgium; ^4^Semiconductor Physics, Department of Physics and Astronomy, KU Leuven, Leuven, Belgium; ^5^MICAS, Department of Electrical Engineering (ESAT), KU Leuven, Leuven, Belgium; ^6^Leuven Institute of Micro- and Nanoscale Integration (LIMNI), KU Leuven, Leuven, Belgium

**Keywords:** single-cell, microbial ecology, optical techniques, electrical techniques, flow cytometry, Raman spectroscopy, CMOS-MEA, impedance flow cytometry

## Abstract

New techniques are revolutionizing single-cell research, allowing us to study microbes at unprecedented scales and in unparalleled depth. This review highlights the state-of-the-art technologies in single-cell analysis in microbial ecology applications, with particular attention to both optical tools, i.e., specialized use of flow cytometry and Raman spectroscopy and emerging electrical techniques. The objectives of this review include showcasing the diversity of single-cell optical approaches for studying microbiological phenomena, highlighting successful applications in understanding microbial systems, discussing emerging techniques, and encouraging the combination of established and novel approaches to address research questions. The review aims to answer key questions such as how single-cell approaches have advanced our understanding of individual and interacting cells, how they have been used to study uncultured microbes, which new analysis tools will become widespread, and how they contribute to our knowledge of ecological interactions.

## 1. Introduction

Single-cell analysis has gained increased significance in microbiology. The idea of analyzing individual cells emerged back in the 19th century, when Antoni van Leeuwenhoek used his self-constructed microscopes to observe microbes for the first time (Lane, 2015). Since then, advances in technology have enabled researchers to investigate individual cells in even more detail, with significant inferences for understanding microbial ecology.

**Figure 1:**
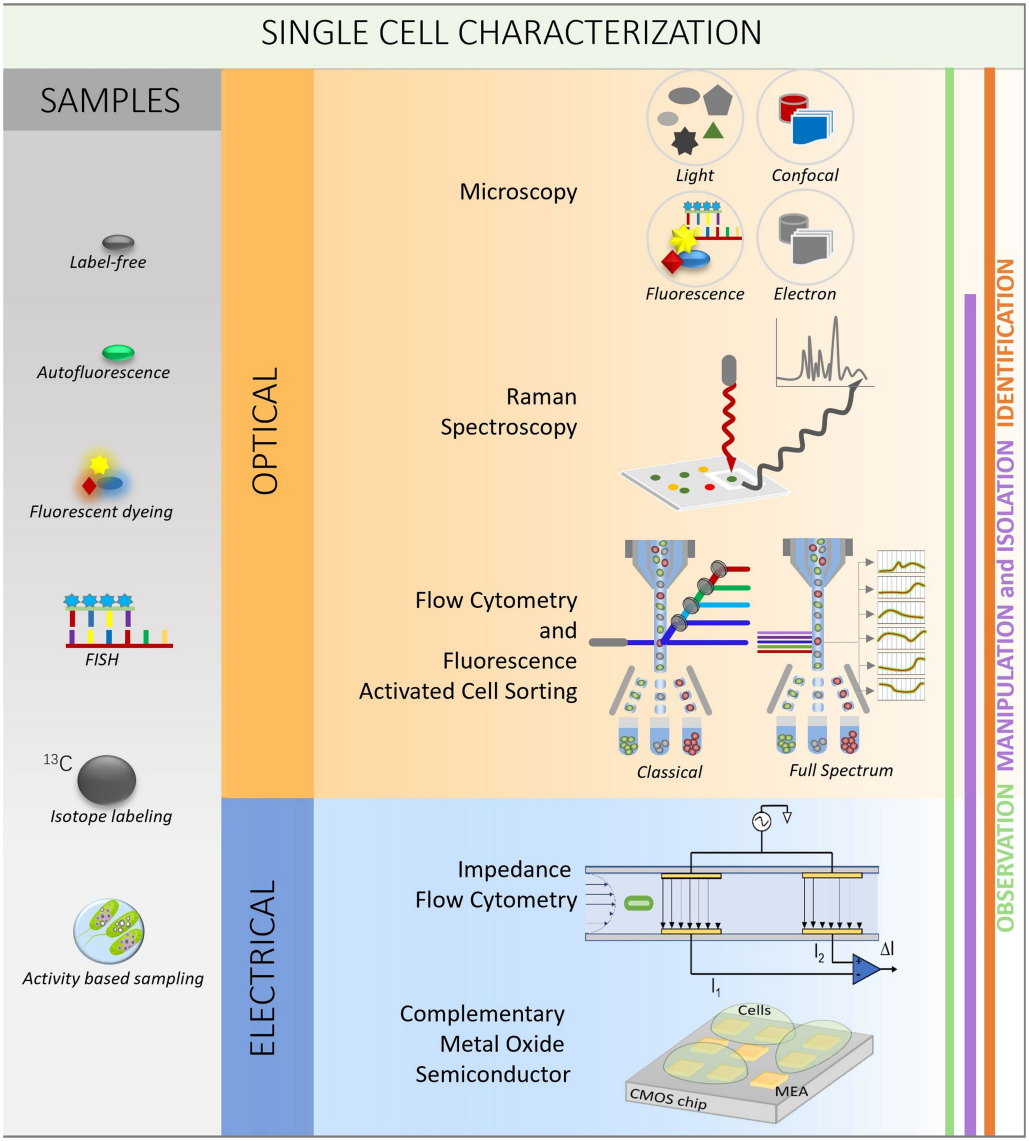
Workflow for single-cell analysis. The left column explains different methods used for sample preparation (gray). The two squares in the middle are showing optical (orange) and electrical (blue) methods to observe (green line on the left), manipulate and isolate (purple line) and identify (orange line) the single cells.

Microbiologists have traditionally studied microbes using culture-based methods (Hugerth and Andersson, 2017). These techniques include the controlled growth of microbial cells in the laboratory to produce pure cultures, that can subsequently be examined using various biochemical and physiological assays. The notion of microbial “VBNC (viable but not culturable) state of bacteria” was inspired by the fact that many microbial species cannot be successfully grown in the lab using culture-based approaches (Marcy et al., 2007). Moreover, cellular heterogeneity is a crucial trait of biological systems because it provides for a broader range of responses to changing environmental conditions. Understanding the information contained inside is critical for developing better models of cell activity, as well as serving as meaningful readouts of population physiology and predictors of response to perturbations (Furst and Francis, 2019). Hence, single-cell analysis, which enables the direct examination of individual cells from environmental samples, has the potential to reveal this VBNC state of bacteria, as well as cellular heterogeneity (Huys and Raes, 2018). Traditionally, the techniques used for microbial communities obtain averaged community traits via bulk analysis of DNA, RNA, or protein from mixed populations (Lane et al., 1985; Stein et al., 1996; Props et al., 2017). Instead of relying on averaged bulk data, researchers can better comprehend microbial communities and their roles by studying individual phenotypic and genotypic traits. Several optical and electrical single-cell technologies exist to observe, manipulate, isolate and identify the single cells (Figure 1).

Next, to single-cell sequencing, some of the most promising methods for single-cell analysis now rely on optical technologies. These enable real-time observation and quantification of individual cells. However, most of the uncultured microbial species make it challenging to research their physiology and interactions with other organisms (Nichols et al., 2008; Chaudhary et al., 2019). Hence, with optical technologies, single-cell analysis has emerged as a valuable tool for exploring the diversity and functional potential of microbial communities because they allow analysis of microbes without the need for culturing.

Single-cell analysis has been extensively employed using optical technologies ever since Robert Hooke published his discovery of cells in *Micrographia* during the 17th century (Hooke, 1665). In the 20th century, new technologies such as fluorescence microscopy, flow cytometry, and Raman spectroscopy emerged, opening up new fields of cell analysis (Laerum and Farsund, 1981; Denk et al., 1990; Puppels et al., 1990). The emergence of the laser had a significant impact on the study of single cells, leading to the development of novel microscopic techniques and flow cytometry (Denk et al., 1990; Bedner et al., 1999). In 2014, Nobel laureates in chemistry were awarded for their work on super-resolution (SR) microscopy, which enables imaging of single cells with resolution beyond the optical diffraction limit (Möckl et al., 2014). Quantitative optical technology advancements for single-cell analysis are anticipated to improve resolution and throughput, leading to enhanced measuring capabilities for single cells.

Due to their capacity to provide real-time, label-free and non-invasive observations (Hedayatipour et al., 2019), electrical and electrochemical methods are gaining popularity as a viable tool for single-cell analysis in microbial ecology. Impedance flow cytometry (IFC), for instance, enables the characterization of a range of biological characteristics of a cell, such as size, viability and biophysical changes in membrane permeability (Spencer et al., 2020). Complementary metal-oxide semiconductor microelectrode arrays (CMOS MEAs), on the other hand, provide the ability to detect and characterize microbial cells or biofilms with an array of parallel sensors (Valente and Demosthenous, 2017). These methods have the ability to open up new avenues for the study of microbial systems and offer insightful information on the physiology and behavior of microbes.

In order to investigate microbial ecosystems, this review emphasizes the theoretical foundations of quantitative optical and electrical single-cell technologies. It supports the blending of known and creative ideas by providing examples from recent research to show how they have been applied successfully. Applications of discussed technologies will focus on microbial cells in liquid suspension mostly since the majority of these techniques are best suited for this sample type. Each approach’s benefits and drawbacks will be thoroughly discussed, and prospective research possibilities will be offered to shed light on the anticipated future tools and procedures. The knowledge gained in these fields can aid in controlling the role of microbes in life. Therefore, we can expect that there will be numerous advancements in the study of single-cell microbial ecology through the use of optical and electrical techniques.

## 2. Microscopy

Since more than a century ago, microbes have been observed and studied using the well-established method of microscopy (Gest, 2004). As it enables researchers to see individual cells’ shape, activity, and interactions with other cells and the environment, it is very helpful for analyzing single cells. Microbial ecology frequently uses microscopy methods to investigate microbial diversity, community structure, and function.

For single-cell investigation, a range of microscopy methods can be utilized, each with unique advantages and disadvantages. One of the most used microscopy methods in microbiology is light microscopy (Reymond and Pickett-Heaps, 1983). It gives excellent spatial resolution and contrast while using visible light to monitor cells, making it suitable for examining the morphology of single cells. A particular kind of light microscopy called bright field microscopy depends on the sample’s inherent contrast, with light passing the sample and diffracting differently depending on the cells’ characteristics (Selinummi et al., 2009). It is simple to use and offers an overall picture of the sample, but it provides little insight of the interior cellular components. Fluorescence microscopy, on the other hand, labels specific cellular structures or molecules with fluorescent dyes or proteins, allowing imaging of biological processes such as gene expression, protein localization, and cell signaling (Kural et al., 2005).

For focused investigation of certain sample components, other microscopy methods including confocal microscopy (Nancharaiah et al., 2007) and electron microscopy (Paddock, 1999; Croix et al., 2005) are utilized. Confocal microscopy offers high-resolution 3D imaging of cells and tissues by combining the benefits of both light and fluorescence microscopy, while electron microscopy provides thorough information on the cellular structure by using an electron beam to see cells at the nanoscale level (van Manen and Otto, 2007). For example, scanning electron microscopy (SEM) may be used to scan samples’ surfaces to examine the structure and morphology of cells (Paddock, 1999; Croix et al., 2005), and transmission electron microscopy (TEM) (Kemmerling et al., 2013) employs an electron beam to produce a high-resolution image of the interior structures of fixed and sectioned cells. Other techniques like atomic force microscopy (AFM) (Li et al., 2019) uses a tiny probe to provide topographic images of the sample’s surface and can scan living cells in their hydrated condition. Raman microscopy employs laser light to look at molecules’ vibrational modes. Raman scattering causes photons from a laser beam that interact with a sample to disperse in various directions (often at a wavelength that differs from the initial laser light) (Schuster et al., 2000).

Combining different microscopic methods to gain more detailed information on a sample has grown in popularity in recent years. Researchers can profit from the advantages of each approach while overcoming their limits by combining them. The blend of fluorescence and electron microscopy is one illustration of a frequently employed set of methods (Li et al., 2017). Researchers may visualize the location of individual molecules within a biological setting and examine their structural arrangement by combining various approaches. Another example is the use of Raman microscopy in conjunction with confocal microscopy, offering high spatial resolution imaging of the sample’s morphology and chemical composition (Gomes da Costa et al., 2019).

Hybrid Confocal Raman Fluorescence Microscopy (van Manen and Otto, 2007), super-resolution microscopy coupled with electron microscopy (Andrian et al., 2021), X-ray microscopy coupled with fluorescence microscopy (XRFM) (Yin and Marshall, 2012) and XRFM with atomic force microscopy (Penner-Hahn, 2013) are further method combinations. These approaches have a wide range of applications, including materials science, biology, and medicine. Overall, the combination of several microscopic methods can be a valuable tool for investigating intricate samples and expanding our knowledge of numerous microscopic processes.

As morphological properties of a culture contain lots of information on cell physiology, *in situ* microscopy devices have been developed to monitor microbial bioprocesses. This enables to monitor single-cell size distribution, which is coupled to automated image analysis based on an artificial neural networks model. Although successfully applied for monitoring yeast, algae and fungi, smaller cells such as bacteria still face practical limitations of resolution (Marbà-Ardébol, 2018; Marbà-Ardébol et al., 2019).

While microscopic analysis of microbial ecology can give excellent resolution, there are significant limitations connected with this method. Assessing the diversity and heterogeneity of natural microbial communities is one of the most difficult tasks (Widder et al., 2016). Another issue is the scarcity of acceptable sample preparation technologies. Environmental samples frequently contain a high concentration of organic and inorganic materials, which might obstruct microscope findings. To overcome this obstacle, researchers must carefully choose and improve sample preparation procedures in order to collect high-quality pictures and correct data on microbial cells. Furthermore, many microscopy techniques need the use of expensive and specialized equipment, as well as substantial knowledge to execute and interpret the results. This can restrict their use and accessibility in particular research contexts. Finally, interpreting microscopy data can be difficult since microbial cells in natural habitats might have a broad variety of morphologies, sizes, and metabolic activities (Widder et al., 2016). Microscopy data must be thoroughly analyzed and interpreted in order to identify and quantify single cells, and this is typically done in conjunction with other analytical techniques. Overall, while microscopy methods give useful information on environmental microbes, their effective application necessitates careful planning of sample preparation, apparatus, and data processing.

In the context of statistical analysis and quick characterization of microbial ecology, proper image processing is crucial. There are several image processing software tools available that allow researchers to extract quantitative data from microscope pictures. This approach has been used to monitor filamentous bacteria present in wastewater treatment plants’ activated sludge (Ang et al., 2019; Campbell et al., 2019). A classification engine for environmental microorganisms has been developed, which uses automated analysis of microscopic images through the application of Deep Convolutional Neural Networks and Conditional Random Fields (Kosov et al., 2018). In a similar vein, a new Low-Cost U-Net (LCU-Net) has recently been proposed for the segmentation of Environmental Microorganism (EM) images. This tool helps microbiologists to detect and identify EMs more effectively (Zhang et al., 2021). In a different study, in order to map the spatial interaction networks within single-cell communities and to evaluate metabolic interactions among them, mathematical modeling was combined with the growth rate measurements of individual cells (Co et al., 2020).

Furthermore, to overcome the difficulties of microbial ecology analysis, microscopic methods can be used with microfluidics. Improved imaging and analysis are facilitated by the use of microfluidic devices, which may be created to provide single cells a controlled microenvironment (de Jonge and Ross, 2011). High-throughput imaging with microfluidic devices enables quick examination of huge numbers of cells. Microfluidics and microscopic methods can work together to manipulate single cells, aiding downstream analysis by allowing cell manipulations like sorting and trapping (Yin and Marshall, 2012). Additionally, cells may be stimulated chemically or physically using microfluidic devices, making it possible to examine how cells react in various environments. Overall, the combination of microscopic methods with microfluidics provides a potent method for the high-resolution imaging and analysis of environmental microbes while resolving some of the difficulties brought on by the complicated and diverse ecology.

In conclusion, despite many advantages, microscopy techniques have certain drawbacks, such as the requirement for sample preparation, the risk of photobleaching and phototoxicity, and the difficulty to examine living cells for lengthy periods of time. Hence, other optical instruments that may overcome these restrictions and give more extensive information about microbial ecology analysis are required.

## 3. Flow cytometry

Flow cytometry (FCM) is a technique used for detecting and counting particles or discriminating particles with specific characteristics (Box 1). Applications of microbial flow cytometry have been around since the late 1970s (Paau et al., 1977), and have mostly been used to assess microbial quantities (Wang et al., 2010; Frossard et al., 2016; Chodkowski and Shade, 2017; Van Nevel et al., 2017; Gryp et al., 2021). However, phenotypic attributes of cells, including size, intracellular complexity, macromolecular composition, viability, vitality, and other properties based on scattered light or fluorescent signals of single cells in a heterogeneous population can be measured in high-throughput as well (Sieracki et al., 1999; Müller and Nebe-Von-Caron, 2010; Hatzenpichler et al., 2020; Singh and Barnard, 2021). It is important to note that certain properties can be obtained directly without further staining, while others require staining procedures. For example, forward scatter (FSC) can be directly related to cell size (Robertson and Button, 1989; Koch et al., 1996), whereas respiratory activity can only be determined through the use of a stain [e.g., 5-cyano-2,3-ditolyl tetrazolium chloride (CTC)] (Sieracki et al., 1999).

**Figure 2:**
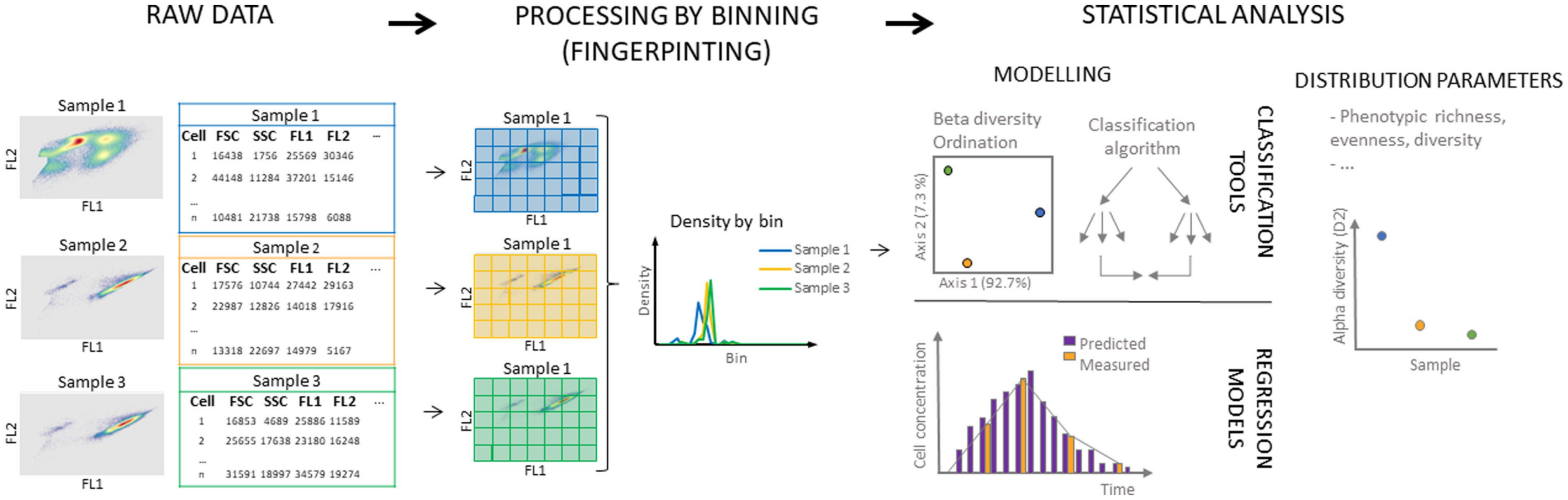
Schematic overview of microbial cytometric fingerprinting and its most common uses. Raw flow cytometry data are obtained from the measurement of the sample (left), and are often displayed in two-dimensional density plots. For each cell, scatter and fluorescence can be measured, leading to multi-parameter data for each individual cell. Following, the cytometric space is divided in bins (middle) and the density of cells in each bin is determined. In this schematic, equal size binning in two dimensions is displayed, but alternative binning approaches in multiple dimensions can be considered. Obtained discretized data (data in bins) can be used for further statistical analysis (right). Distribution parameters such as richness, evenness, and diversity can be calculated as well as between diversity [for example non-metric multidimensional scaling (NMDS) or Principal Coordinate Analysis (PCoA)]. Data can also be used to train classification algorithms and regression models.

Cells are typically visualized in single parameter histograms or dual-parameter dot plots where cells with similar characteristics will be located closer to each other. Depending on the instrument and chosen staining procedure, up to 64 features per cell can be measured (Büscher, 2019). Moreover, the obtained data for the different parameters of cells in the sample can be used to construct a cytometric fingerprint of the microbial community (Figure 2) (De Roy et al., 2012; Koch et al., 2013a,b; Props et al., 2016). It involves dividing the cytometric space into regions (i.e., bins) in which cell densities are recorded. This results in multivariate distributions of the microbial community that can be used for statistical analysis including the determination of ecological parameters and predictive modeling (Rubbens and Props, 2021). Thus, the fingerprint represents the phenotypic microbial community state and can be used to study phenotypic heterogeneity in microbial populations (Props et al., 2016). The use of adaptive binning approaches, such as PhenoGMM (clustering algorithm based on Gaussian Mixture Models), shows potential to improve the discriminative power of the cytometric fingerprint and further its possible applications (e.g., as a diagnostic tool) (Amir et al., 2013; Sgier et al., 2016; Liu et al., 2019; Rubbens et al., 2021).

**Figure 3:**
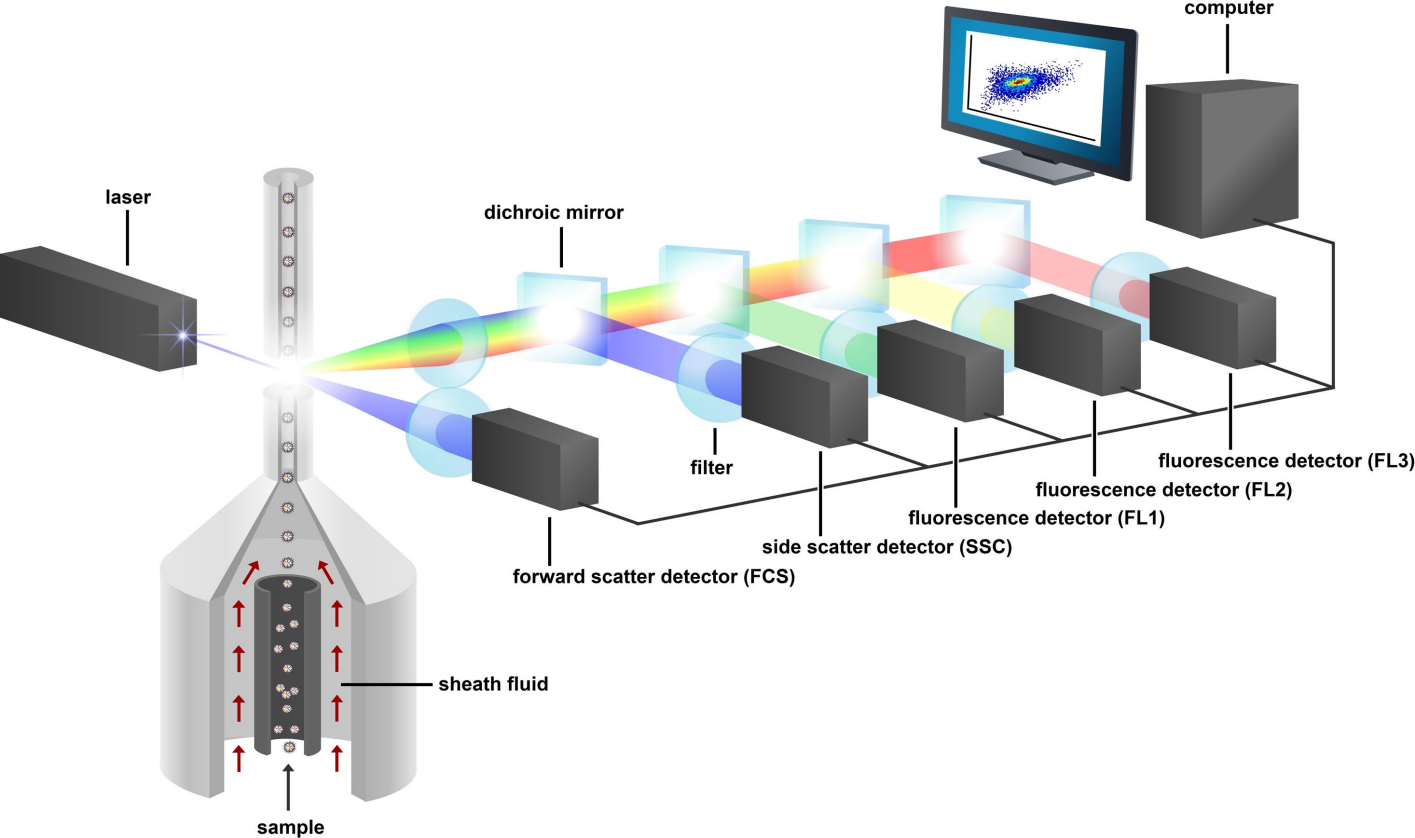
Schematic representation of a flow cytometer. The fluidic system compromises the sample line with sheath fluid. The laser, dichroic mirrors, and filters make up the optical system, the PMT detectors (FCS, SSC, FL1, FL2, and FL3), and the computer make up the electronic system. Figure adapted from Rubbens and Props (2021).

Props et al. showed that estimates of microbial diversity dynamics can be made using these fingerprints, and that obtained alpha diversity profiles strongly correlate with alpha diversity profiles based on 16S rRNA gene amplicon sequencing (Props et al., 2016). Additionally, microbial flow cytometry has been used to detect disturbances in microbial ecosystems (Hammes et al., 2012; Besmer and Hammes, 2016; Van Nevel et al., 2017; Props et al., 2018). Besmer et al. constructed instrumentation for the automation of microbial flow cytometry, referred to as real-time or online flow cytometry (Besmer and Hammes, 2016). They illustrated that real-time flow cytometry was able to detect disturbances in microbial ecology for both engineered and environmental ecosystems. These disturbances would probably be missed when relying on more infrequent sampling (Thyssen et al., 2014). Since then, real-time flow cytometry has been applied to tackle numerous problems, such as follow-up of a full-scale water treatment plant (Sadler et al., 2020), monitoring of quantitative and diversity population dynamics of microalgae (Haberkorn et al., 2021), automated detection of changes in microbial communities (Thyssen et al., 2014), and detection of microbial instability in the drinking water network (Favere et al., 2020).

Over the last few years, the cytometric fingerprint has been used for classification purposes in a wide range of applications as well. Examples include the discrimination of different brands of natural mineral water (De Roy et al., 2012), microbial strain differentiation of Lactobacilli (Buysschaert et al., 2018), and the prediction of Crohn’s disease (Rubbens et al., 2020) using fecal samples. In general, the classification includes the use of algorithms, such as random forest classifiers or artificial neural networks (Rubbens et al., 2020; van de Velde et al., 2022).

However, challenges remain if the use of microbial flow cytometry is to expand. First, microbial cells need to be in a planktonic state to be analyzed on a flow cytometer. This means that bacteria growing in a sessile manner (e.g., biofilms) need to be disrupted and brought in suspension to be effectively measured, which often causes extensive sampling and sample preparation protocols (Kerstens et al., 2015; Sgier et al., 2018; Brown et al., 2019; Chatzigiannidou et al., 2020).

Second, most applications of flow cytometry are dedicated to the study of mammalian cells (Quixabeira et al., 2009; Cossarizza et al., 2021). This leads to progress regarding instruments and research being driven by these applications. In immunophenotyping large panels of antibodies are regularly used to assess different cell characteristics (Mair and Prlic, 2018; Post et al., 2018). However, microbial cells display different characteristics and need alternative sample manipulation methods. Most microbial cells are much smaller, and the size range is much larger, ranging from 0.2 μm to 500 μm (Levin and Angert, 2015). This may result in parts of, or whole measurements, being close to the detection limit of the instrument and implies the need for effective staining procedures. Additionally, microbial communities often display large heterogeneity within a sample as a consequence of complex taxonomic and phenotypic community structure (Müller and Nebe-Von-Caron, 2010). Therefore, the use of multicolor panels involving antibodies is less suited for the study of microbial ecosystems. More suited and widely applied are single or dual staining methods that mark general phenotypic properties (e.g., SYBr^®^ Green I (SG) for nucleic acid content, propidium iodide (PI) for membrane integrity) (Buysschaert et al., 2016; Koch and Müller, 2018). Advances in new cell staining protocols for microbiota have been hampered by differing staining efficiencies between microbial taxa, as well as issues with fluorescence stability (Buysschaert et al., 2016). The result is lower dimensional data compared to data obtained from mammalian cells.

Another bottleneck in current microbial flow cytometry is sample preservation up until flow cytometric measurement. Samples are sometimes fixed to analyze later on, and these fixatives often induce morphological and functional changes (Troussellier et al., 1995; Rocha et al., 2018; Zhu et al., 2021). Especially protocols involving markers for membrane integrity are affected by this phenomenon (Falcioni et al., 2008; Habtewold et al., 2016). This highlights the need for the development of adequate sample preservation protocols and some advancements have been made already. For example, Cichocki et al. showed that PFA/ethanol fixation is suitable for the preservation of a microbial community when using DNA stains DAPI or SG I for analysis (Cichocki et al., 2020). Additionally, staining procedures involving incubation times may pose a challenge as bacterial cells grow, multiply and can change in metabolism very fast (Gibson et al., 2018).

**Box 1**

A flow cytometer consists of three major systems: a fluidic system, an optical system, and an electronics system (Figure 3). The fluidic system allows particles to pass by a laser one by one using hydrodynamic focusing. Particles in suspension are injected into a pressurized stream of sheath fluid. This results in a flow of particles with a relatively large separation compared to their diameter. Next, the stream containing the separated particles passes through a laser where light is scattered and possible present fluorophores are excited. The point where the laser interacts with the particle is referred to as the interrogation point. Scattered light in the forward and sideward direction and fluorescent light are directed to one of several photomultiplier tubes (PMT) by a series of filters and mirrors. These filters determine the wavelength of the light that reaches the PMT. In turn, the PMTs convert the optical signal to an electronic signal, which is then amplified and sent to a computer. The computer provides software for data analysis of collected data. The PMTs and the computer make up the electronic system of the machine (Shapiro, 2005). The speed at which flow cytometers can measure particles keeps increasing as the technology advances and speeds of 10,000 particles/s and higher are now common (Bendall et al., 2012; Delmonte and Fleisher, 2019). Detection limit in terms of particle density is dominated by errors in experimental procedure and not by the sensitivity of the instrument itself. This is the result of the instrument measuring signals for each individual particle (Roederer, 2008). Reports show detection limits of ca. 200 microbial cells/mL for drinking water, and between 1 and 1,000 CFU/mL for bacteria in culture medium (McHugh and Tucker, 2007; Hammes et al., 2008; Karo et al., 2008). Considering particle size, the limit of detection is dependent on the instrument as well as on the fluorescent properties of the (labeled) particle and usually ranges about 100–200 nm (Steen, 2004; Hu et al., 2018; Botha et al., 2021).

**Figure 4:**
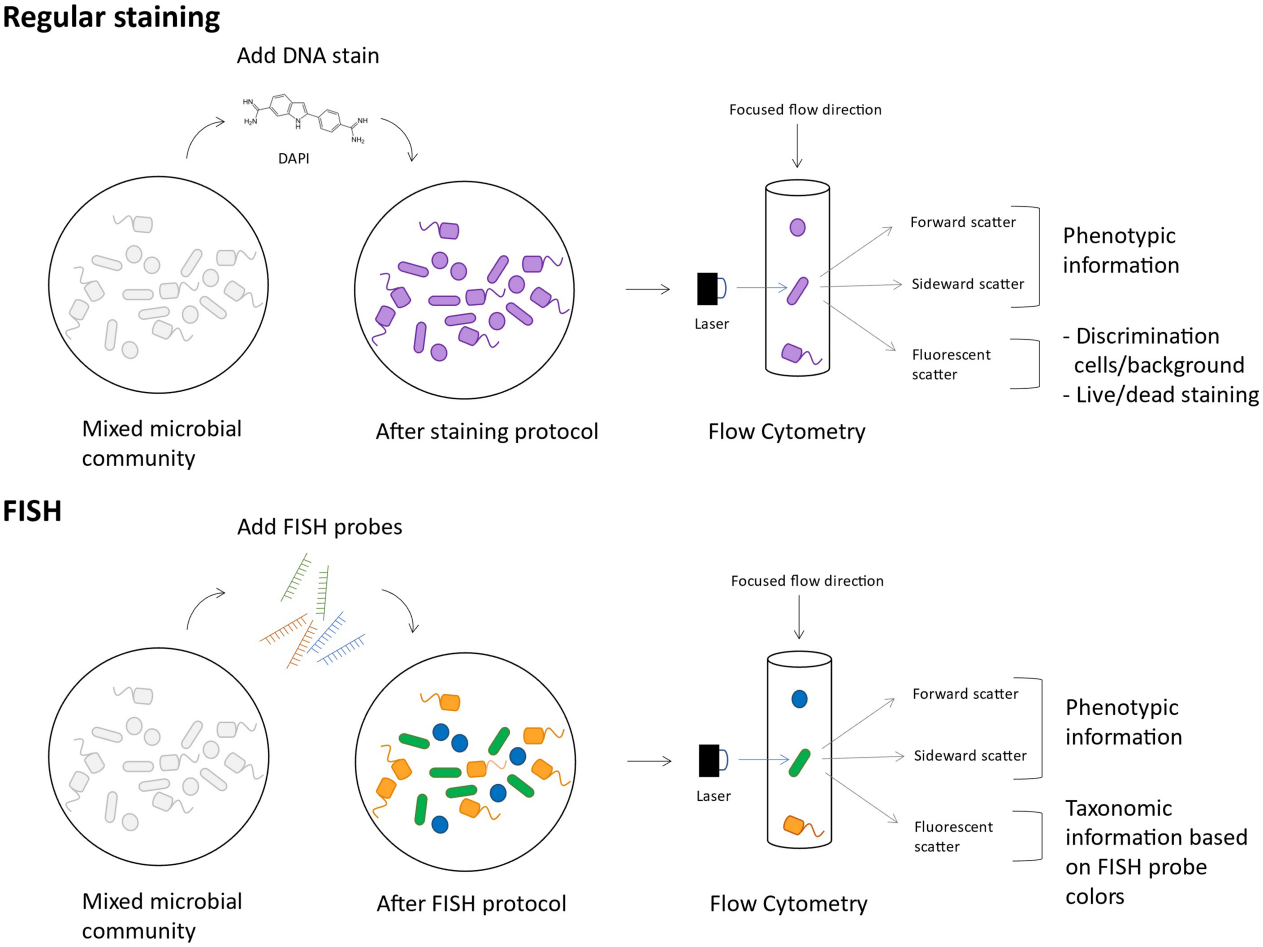
Conceptual figure on FlowFISH. The main difference between regular staining and FlowFISH is that in the latter case, cells are differently fluorescently labeled according to their taxonomy and thus contain taxonomic information in their fluorescent scattering.

When flow cytometry is combined with Fluorescent *In Situ* Hybridisation (FISH), abbreviated as flowFISH, it is possible to taxonomically discriminate groups of bacteria or target specific genera, based on fluorescent labels (Figure 4) (Rigottier-Gois et al., 2003; Rochet et al., 2004). The FISH technique is based on pioneering hybridization experiments by Gall and Pardue (1969), and has developed into a widely used technique that utilizes fluorescent DNA probes to bind specific RNA sequences (O’Connor, 2008). This method has applications for microbial ecology studies wherein the growth dynamics of a specific species or genus can be followed in a microbial community. However, also on higher taxonomic levels it can be useful to follow which classes or orders of bacteria dominate when they are exposed to different types of stress. Moreover, as the probes are hybridized on RNA, even estimations of transcriptional activity can be made. To increase the fluorescent signal in cells with low metabolic activity, FISH can be combined with catalyzed reporter deposition (CARD-FISH), that amplifies the fluorescent signal (Kubota, 2013), and be measured with flow cytometry. Although CARD-FISH requires a solid support, an optimized detachment protocol ensured 85.7% of cells were successfully measured (Manti et al., 2011).

**Figure 5:**
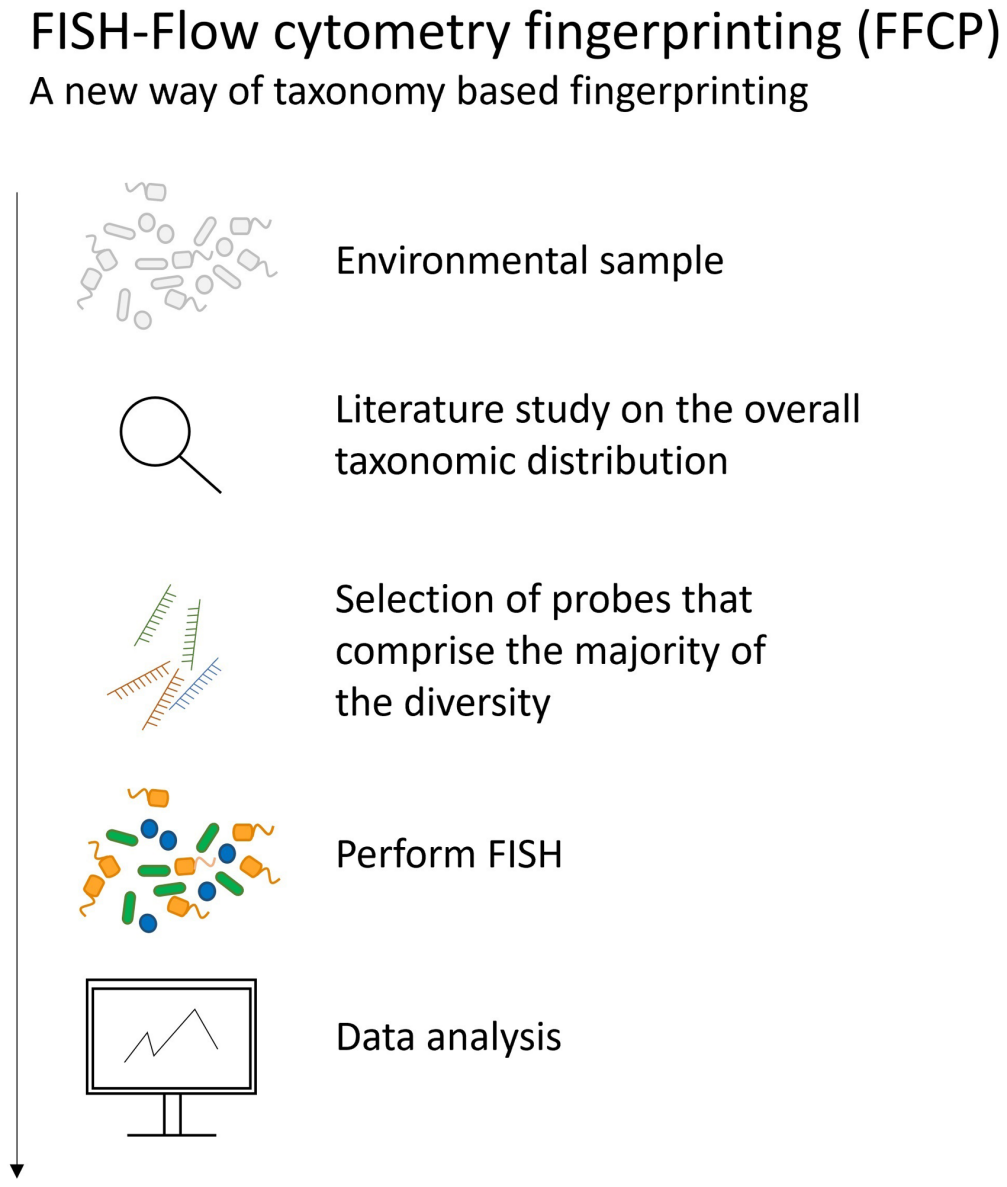
The pipeline followed when performing FISH-flow cytometry fingerprinting of microbial communities.

Recently, the combination of taxonomic (FISH) and phenotypic (FCM) information was used to obtain an integrated community fingerprint (Figure 5). As these data contain multiple additional fluorescence parameters, next to the conventional cell parameters, the information in the fingerprint increases, which can enhance the statistical diversity analysis. Furthermore, it is hypothesized that the diversity analysis of a microbial community containing FISH labeled cells correlates better to the diversity analysis obtained from 16S rRNA gene sequencing data than general DNA stained cells. The use of multiple lasers and differently tagged probes have the potential to increase the resolution of the fingerprint. This method can be a valuable alternative to current techniques such as qPCR to answer some ecological questions concerning the presence or the abundance of a certain, or multiple species. Next to taxonomic tagging within a microbial community, also translational tagging can be done. For example, bio-orthogonal non-canonical amino acid tagging (BONCAT) allows to label only the active protein-producing cells (Lindivat et al., 2020). This was for example applied to explore vitality of single cells after UV irradiation and heat treatment (Lindivat et al., 2021).

To increase the spatial resolution of flow cytometry and extract the exact cell size, Image Flow Cytometry can be used. It combines high-throughput flow cytometry with single-cell imaging by microscopy. The images help to distinguish differences between cells, debris, and aggregated cells and thus and facilitate gating decisions. The main limitation of image flow cytometry is that large amounts of data are generated in minimal amounts of time and the cell images produced by image flow cytometry are much more complex to analyze. Successful applications of this technique can be found in the field of virology and more specific in the study towards virus-host interactions (Han et al., 2016; McClelland et al., 2021). Sorting real-time deformability cytometry is a microfluidics technique that uses brightfield images to sort out cells with clear morphological differences. The images are analyzed by a deep neural net to make the sorting decisions (Herbig et al., 2022).

To facilitate multicolor applications with fluorophores with similar emission peaks but off-peak signatures, spectral flow cytometry can be used. It is a technique in which full spectral measurements are made across all lasers, instead of only identifying the peak emission by conventional flow cytometry, by the use of a larger number of detectors with narrow band-pass filters (Nolan and Condello, 2013; Ferrer-Font et al., 2020). However, for successful application fluorescent labels should be stable and have fixed emission spectra (Liu et al., 2022). Examples of successful application of this technique can be found in immunotherapy research (Bonilla et al., 2021), immunophenotyping assays (Ferrer-Font et al., 2020), or *in vivo* cellular movement (Futamura et al., 2015). The technique has not been applied to microbial samples yet, although it may be useful when working with complex panels of fluorescent labels or with auto fluorescent cells. With spectral unmixing approaches, an intrinsic cell auto fluorescence signal can be separated from extrinsic fluorescence labels, and in complex combinations these extrinsic labels can be separated from each other as well. For other applications, it is however important to consider whether the spectral information provides significant advantages to conventional flow cytometry (Nolan and Condello, 2013).

## 4. Fluorescence activated cell sorting

Phenotypic or taxonomic (RNA-labeled) subpopulations identified through flow cytometry can be sorted out with fluorescence activated cell sorting (FACS). The cell suspension is put in a narrow stream wherein the cells are separated cell by cell after which a vibration mechanism breaks up the stream in individual droplets with a high probability of one cell per droplet. Through electrical charging of the droplets, they can be broken off from the stream and sorted in separate recipients (Naeem et al., 2017).

**Figure 6:**
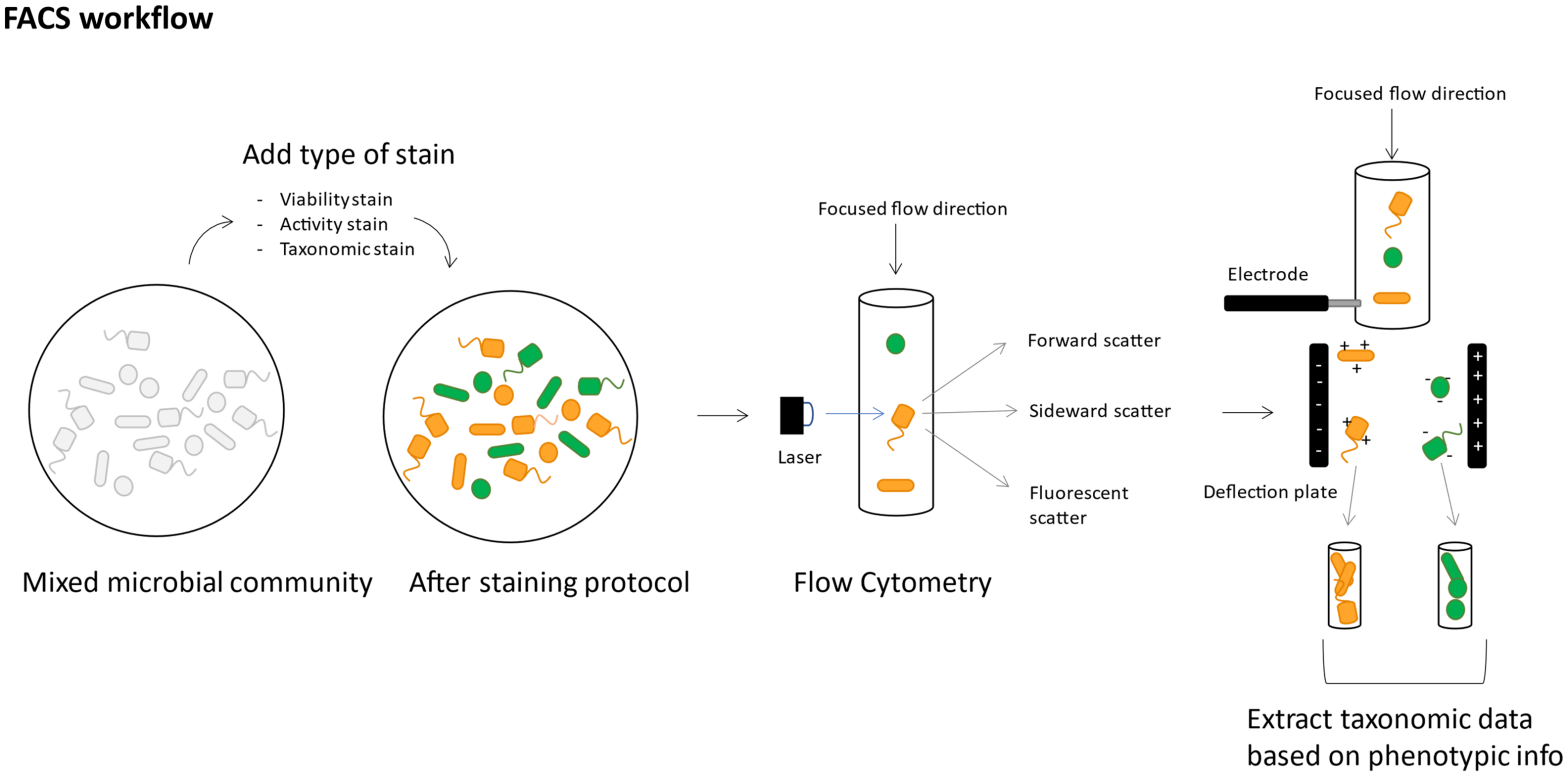
Conceptual figure of a suggested FACS workflow, showing how it can allow to use phenotypic data as a basis for taxonomic data extraction.

By specific cell-staining, like intact/damaged staining, activity-based staining or FISH, populations can be sorted out based on this specific attribute (i.e., intact, damaged, active, non-active, belonging to a specific taxon) (Figure 6). For example, nucleic acid viability staining methods like SYBr^®^ Green I (SG) combined with propidium iodide (PI) allow to differentiate between cells in a different state (intact/damaged) and consequently sort out the specific populations (SG+, PI+, SGPI+, SGPI-) and use this to identify only the intact bacteria participating in the functioning of the microbiome (Bellali et al., 2021). Bacteria in the human microbiome are remarkably physiologically heterogeneous when comparing damaged versus intact populations (Ben-Amor et al., 2005). This could have serious implications in medical applications, where the viable microbial population is the most relevant population when looking into managing the microbiome. FACS based on translationally active fluorescently labeled cells like bio-orthogonal non-canonical amino acid tagging (BONCAT) (Couradeau et al., 2019) allows to investigate which bacterial species are performing a certain specific ecological task and when they are performing it through time, without interrupting their native ecosystem (Du and Behrens, 2021). Finally, FACS can also be performed after FISH probing, investigating fine-scale differences of a gene homolog in a genus/family (Kim et al., 2010). Recently, efforts on live bacterial cell sorting have significantly improved the success rate of cultivation of not (yet) culturable bacteria (like certain soil bacteria) by first sorting out viable cells (Espina, 2020). Moreover, Batani et al. proved that it was possible to cultivate bacteria after labeling them with fluorescent RNA probes and sorting them out (Batani et al., 2019).

The DNA of sorted bacterial subpopulations can subsequently be extracted and used for 16S rRNA gene amplicon sequencing (Reichart et al., 2020; Heyse et al., 2021) or even whole-genome sequencing (Rinke et al., 2014) and (targeted) metagenomic sequencing (Grieb et al., 2020). This allows to potentially couple back phenotypic traits determined by flow cytometry with specific taxonomic groups, as was performed by Heyse et al., where the authors were able to sort out specific phenotypic groups of shrimp cultivation water microbial communities and link it to specific bacterial taxonomic groups (Heyse et al., 2021). When this data is used for predictive modeling, it allows to capture taxonomic information within the phenotypic data obtained through flow cytometry.

Similar to FCM, FACS was originally developed for mammalian cell handling and the cytometer part of the FACS suffers of the same limitations. Moreover, FACS results in very low abundance samples which can make subsequent molecular analysis difficult, sensitive to bias and sensitive to potential (cross-)contamination (Brandt and Albertsen, 2018).

## 5. Raman spectroscopy

**Figure 7:**
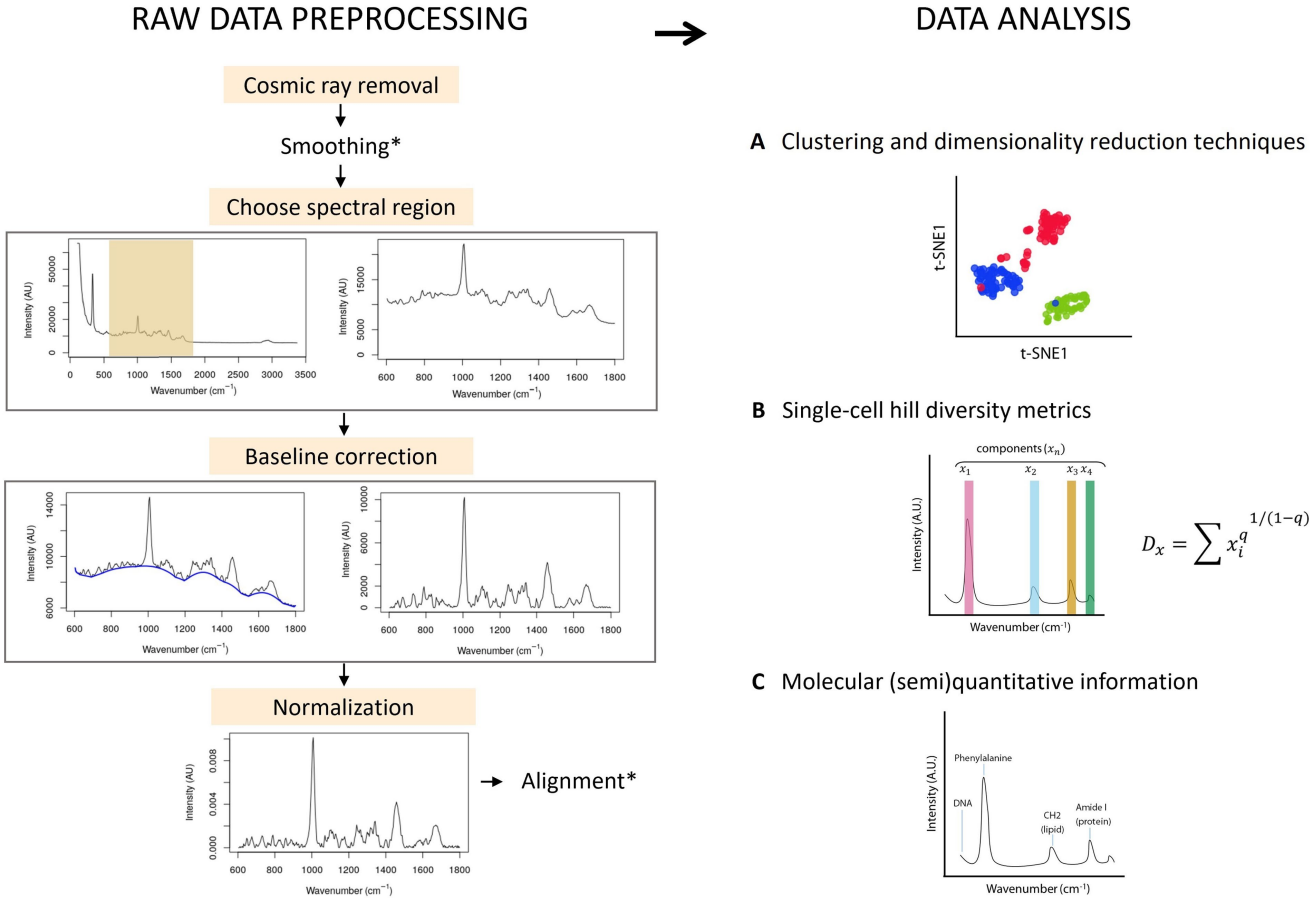
Raw data preprocessing of Raman spectra & data analysis. Left: First, the spectra are baseline corrected and normalized. Smoothing and alignment steps can be included. However, smoothing can erase potentially relevant information and should be carefully considered. Similarly, alignment can produce faulty spectra by displacing the signal and thus needs to be used with care. Right: Information that can be obtained with single-cell Raman spectra of cells: **(A)** The spectrum of individual cells can be plotted using clustering and/or dimensionality reduction techniques. **(B)** The peaks of the Raman spectra correspond to a different metabolite or a combination of metabolites, called here components (x). The intensity of the signal of each component can be normalized by the sum of all intensities, and this information can be then used in the Hill equation. The order of diversity (q) can be 0, 1 or 2, meaning that the richness, evenness or both richness and evenness are taken into consideration in the metric. **(C)** The information from the spectral peaks correspond to one or multiple molecules, and can be used (semi)quantitatively.

Most ecological studies rely on marker gene expression, metagenomics or transcriptomics to describe the functionality of microbial populations. Raman spectroscopy presents an opportunity to describe single-cell diversity with or without labels, and describe phenotypic changes and metabolic information in a (semi) quantitative way. Raman spectroscopy records spectra that result from the inelastic scattering of photons from a molecule. The result is a spectrum with several peaks that correspond to a particular chemical bond and their vibrations. Raman spectra can be used as a fingerprint to identify bacteria (Goodacre et al., 1998; Willemse-Erix et al., 2009; Kusić et al., 2014) or to obtain semi-quantitative information about the components of the cell (Butler et al., 2016), that can be quantitative if a standard for the molecule(s) of interest is made (Figure 7) (Lowery et al., 2017).

Conventional Raman spectroscopes are based on Stokes Raman scattering, which is relatively weak as only 1 in 10^6^–10^8^ photons undergo inelastic Raman scattering (Chisanga et al., 2018). This makes obtaining the Raman spectra of a single-cell time consuming compared to other techniques (about 30 s per cell). To reduce the analysis time, metallic nanoparticles can be used. When the laser excites these nanoparticles, an enhanced light field is created, and the Raman signal of the molecules close to this field is enhanced (Pilot et al., 2019). These metals can be used in suspension, on a surface [surface-enhanced Raman spectroscopy (SERS)), or the tip of the scanning probe (tip-enhanced Raman spectroscopy (TERS)]. These techniques increase the Raman signal by 10^6^–10^14^ (Lombardi and Birke, 2009), allowing to scan cells in 1–3 s (Liu et al., 2016). Another way to enhance the Raman signal is to measure coherent anti-Stokes Raman spectroscopy (CARS). This technique uses two laser beams to enhance the Raman signal, increases the signal-to-noise ratio, and allows to use Raman spectroscopy at the sub-micron scale (Song et al., 2016).

Several excitation wavelengths can be used in Raman spectroscopy. Since the Raman scattering intensity is inversely proportional to the fourth power of the excitation wavelength, the higher the excitation frequency, the higher the Raman signal (Tuschel, 2016). UV has a high frequency and thus gives a high Raman signal; however, its radiation can damage the sample. Also, fluorescence occurs mostly when exciting with visible light, therefore choosing a laser in the near infrared can suppress this effect providing a good signal-to-noise ratio (De Gelder, 2008).

Raman spectroscopy is non-destructive, and can be coupled with a sorting system to sort out single cells for cultivation or molecular analysis [Raman-activated cell sorting (RACS)]. The cell isolation can happen in a solution using optical tweezers to trap the individual bacteria (Raman tweezers), with a microfluidic chip (microfluidic based RACS) or on a surface (Raman-activated cell ejection or RACE) (Song et al., 2016). Raman tweezers can be used in combination with a microfluidic system to move the bacteria of interest into a special reservoir for further evaluation. Lee et al. used this technique in cells labeled with isotopes, and sorted 3–8 cells per min (Lee et al., 2019). Microfluidic RACS is a faster alternative that can sort between 5 and 100 cells per sec. The sample needs to be in an aqueous solution, and the cells will pass through a laser one at a time. This technique is analog to FACS, or fluorescence-activated activated sorting, although FACS can measure thousands of cells per second (Song et al., 2016). RACE allows to sort in a non-aqueous sample, such as a biofilm, a tissue sample or a solid surface. In this method, laser pulses pass through a transparent substrate onto a light-absorbing layer (such as water) to disintegrate the layer (evaporate the water) and generate energy to eject the cell. The process takes about 1 s per cell (Wang et al., 2013).

The information of the Raman spectra can be used to observe the physiological state of a cell, and determine the production of a certain biomolecule in a (semi)quantitative way. This can be done in unlabeled bacteria (Teng et al., 2016), or using isotope probing (Wang et al., 2016). For instance, it is common to study the production of unlabeled compounds that have a strong Raman signal, such as chlorophylls, carotenoids and other pigments (Jehlička et al., 2014). Also, labeled molecules such as ^13^C, ^15^N or deuterium can be used to study, respectively, the carbon or nitrogen metabolism or the metabolic rate in natural or synthetic communities (Berry et al., 2015; Muhamadali et al., 2015). Isotope probing can be coupled to cell sorting to further characterize cells that have a certain metabolism or produce a specific molecule. For example, Jing et al. sorted a natural community from the ocean based on the CO_2_ fixation capacity of single cells, and then sequenced these subpopulations. This experiment resulted in the finding of new CO_2_ fixation pathways (Jing et al., 2018).

The Raman fingerprint of cells is often used to identify what strain they belong to. In the public-health field, this is useful to detect pathogenic bacteria. For example, Kearns et al. have developed an assay to trap and identify multiple bacteria using SERS to detect food poisoning (Kearns et al., 2017), and van de Vossenberg et al. have used it in drinking water to discriminate between Legionella strains and between *E. coli* and coliform strains (van de Vossenberg et al., 2013). Strain identification is also useful in armed forces operations, to identify potential bioweapons (Pearman and Fountain, 2006), or in space missions. This tool is a good candidate as samples do not need to be treated or labeled, and the device does not need to contact the studied rock, diminishing the risk of contamination. Additionally, Raman spectroscopy can be used on suspended cells or to study biofilms.

Raman spectroscopy can be used to identify the microbial phenotypes of single cells using clustering algorithms that allow discriminating cells from the same population that have been treated with different stressors such as alcohol, metals, antibiotics and starvation (Zu et al., 2014; Teng et al., 2016; García-Timermans et al., 2020; Tanniche et al., 2020) or that have been cocultured with other bacteria (Heyse et al., 2019). For instance, this is a powerful tool to predict the functional class of an unknown antibiotic, identify individual antibiotics that elicit similar phenotypic responses (Athamneh et al., 2014) and determine the antibiotic susceptibility of bacteria (Novelli-Rousseau et al., 2018). On the other hand, phenotypic differences between single cells can be calculated by applying the Hill diversity framework to the Raman spectra. This method was developed by Garcia-Timermans et al., that compared *S. cerevisiae* subpopulations with a high or low expression of a stress reporter (García-Timermans et al., 2020). Using Hill numbers, it was found that the stressed subpopulation had a higher single-cell phenotypic diversity than the non-stressed.

The use of Raman spectroscopy presents several challenges. First, there can be small shifts from one instrument to another when measuring the same spectra. For instance, the 1,009 cm^−1^ region from phenylalanine has been reported by De Gelder et al. in 1004 cm^−1^ (De Gelder et al., 2007) and by Zhu et al. in 1005 cm^−1^ (Zhu et al., 2011). It is important to take this into account in the experimental setup, analyzing a reference spectrum, and aligning the spectra if necessary in the data processing. Secondly, microbes are complex systems and it is sometimes difficult to disentangle the Raman spectra and define what compound(s) peaks correspond to. Thirdly, some compounds have a greater Raman intensity and are over-represented in the spectra (for example aromatic rings), while others do not show up. Therefore, although Raman spectroscopy is quantitative, this capacity can only be used to compare the same peak(s) amongst samples. Finally, multiple databases describe different Raman wavelengths to identify the same molecules.

## 6. Electrical techniques

Electrical and electrochemical techniques utilize a set of electrodes in contact with the cell medium to measure and apply electric signals. Cells in the medium influence the electric signals and alter the response to electric stimulation. These changes in the signal can be measured and related to cell properties such as size, viability, cell activity, etc. (Xu et al., 2016). The use of electrical analysis techniques has three main advantages over optical methods. Firstly, the devices can be miniaturized and mass-produced, as the history of CMOS (Complementary Metal-Oxide-Semiconductor) scaling has proven (Bohr and Young, 2017). Secondly, these techniques do not require large and expensive components such as lenses or other optical equipment (Hosseini et al., 2023); thirdly, they are label-free, facilitating real-time measurements for continuous analysis. For these reasons, electrical single-cell analysis devices have the potential to become cheaper and smaller than current existing commercial devices and even have the potential to be used as portable single-cell analysis tools. Despite these advantages, electrical techniques usually suffer from a lower sensitivity compared to more established optical methods (Gökçe et al., 2021). Furthermore, many electrical techniques are still in an early stage of development, with only a limited number of *in situ* applications in a bioprocess reported in the literature. Two of the most promising techniques for electrical single-cell analysis devices are discussed here: Impedance Flow Cytometry (IFC) and CMOS Micro Electrode Arrays. Other electrical techniques have been successfully used for microbial single-cell analysis such as dielectrophoresis (DEP) and electrorotation (ROT) but are not further discussed here. Reviews of these techniques can be found in prior work (Li and Anand, 2018; Henslee, 2020; Duncan and Davalos, 2021).

**Figure 8:**
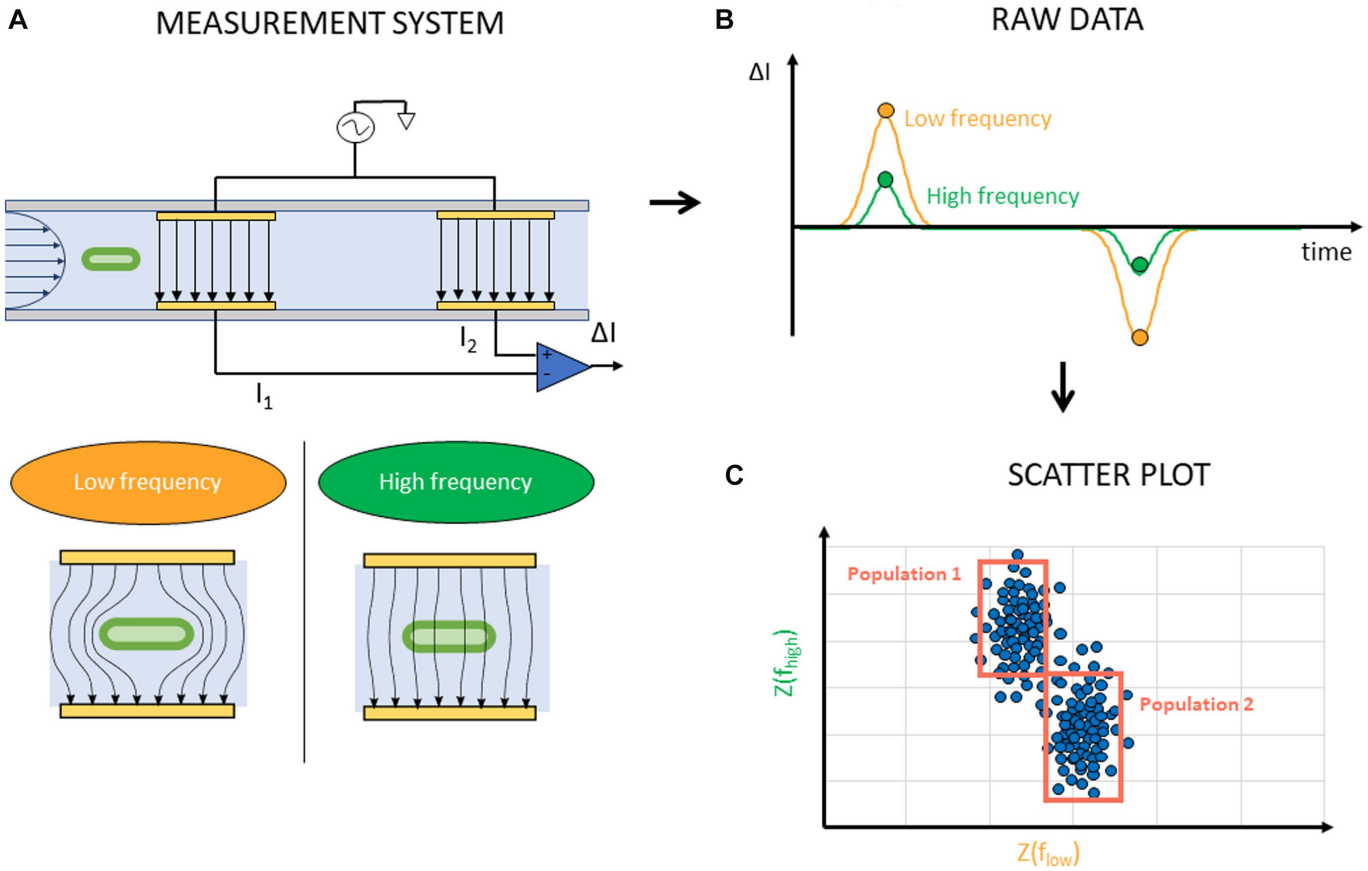
Schematic representation of IFC experiment and data acquisition. **(A)** An AC voltage is applied to the top electrodes in the microfluidic channel at a high and low frequency. The electric field around a cell between electrodes illustrates the frequency dependency. **(B)** A differential current is measured when a cell passes between the electrode pairs. **(C)** From this measurement, the impedance at the two measured frequencies Z(f_high_), Z(f_low_) of each cell is extracted and presented on a scatter plot where gating of cell populations is possible.

Like other FCM devices, IFC devices are comprised of a microfluidic channel where cells are focused in one line. Instead of optically measuring cells, they use micro-sized electrodes along the microfluidic channel to measure the electrical properties of cells in the channel. The principle of IFC is based on the Coulter machines, frequently used for cell counting (Gawad et al., 2001). These machines measure changes in electrical resistance between two electrodes using a Direct Current (DC) signal. On the other hand, IFC systems use Alternating Current (AC) signals to measure the electrical impedance of cells at one or more excitation frequencies and use a more complex arrangement of the electrodes. A differential arrangement of four electrodes is commonly used (Figure 8A). Two electrode pairs, each consisting of one top and one bottom electrode, are placed along the microfluidic channel. A voltage signal is applied to each pair’s top electrode resulting in currents I1 and I2 flowing from the top to the bottom electrodes. The currents I_1_ and I_2_ are subtracted from each other, and the differential signal ΔI is measured. An empty channel results in zero differential current. A current is measured only when a cell passes between one of the electrode pairs (Figure 8B). AC signals provide more information about the cell than using a DC measurement since different cell structures dominate the electrical impedance depending on the frequency. At low frequencies, generally below 1 MHz, the cell membrane forms an insulating layer around the cell cytoplasm and blocks the electric current from passing through its inner volume. The measured signal in this frequency range is therefore related to the volume of the cell. At higher frequencies, generally above 1 MHz, the polarization of the membrane dominates the impedance. At even higher frequencies, above 20 MHz, the membrane seizes to polarize and the electric current passes through the inner cell volume and cell cytoplasm properties dominate the measurement (Honrado et al., 2021; Hosseini et al., 2023). Typically two frequencies, a high and low one, are applied at the same time. From ΔI the cell impedance at these two frequencies Z(f_high_), Z(f_low_) is extracted for each cell and this data is then shown in a scatterplot (Figure 8C). The impedance technically consists of two parts, a magnitude and a phase component. Either of them can be used as the axis for the scatterplot.

IFC was first utilized to detect larger eukaryotic cells (Ayliffe et al., 1999), but recent advancements have improved the sensitivity and opened the way to detect bacteria. Early works reported differentiation between bacteria (*E. coli*) and 1 μm or 2 μm beads (Bernabini et al., 2011; Haandbæk et al., 2014). More recent IFC systems were able to make a distinction between different bacterial strains. Using a measurement frequency of 8 MHz, where electrical properties of the membrane and cytoplasm influence the impedance, Gram-negative *E. coli* and Gram-positive *S. aureus* were successfully distinguished (Clausen et al., 2018). The same group later reported differentiation between live and dead *E. coli* cells (Bertelsen et al., 2020). They noted that their measurement allowed for differentiation between various methods of cell inactivation since heating, ethanol treatment, and autoclaving were observed to induce distinguishable alterations in the cell structure. IFC’s label-free attribute permits real-time monitoring of population dynamics. Spencer et al. demonstrated this by measuring the gradual change in impedance over time as a *K. pneumonia* population responding to an antibiotic (Spencer et al., 2020). Medical diagnostics can also benefit from the use of IFC. Moore et al. reported a device which was able to detect *C. difficile* spores, responsible for *C. difficile* infection (CDI) (Moore et al., 2020). Their device significantly improved the detection time of the 0.5 μm spores compared to the typical method of measuring CFU. Recently, several start-up companies have released commercial IFC systems for use on bacteria. The system provided by *Amphasys AG* (^©^ 2022 Amphasys AG) allows for live/dead differentiation of bacteria larger than 2 μm. *SBT Instruments* (SBT ^©^ 2022) sells a portable tool for bacteria enumeration.

IFC devices have a high throughput of (~10^3^ cells/s) (Chen et al., 2015), which is slower than but close to the throughput of a flow cytometry device (~10^4^ cells/s) (Bendall et al., 2012; Delmonte and Fleisher, 2019). From a technological perspective, the challenge is the short time interval that cells are available to be measured, which limits accuracy and the number of frequencies that can be probed. Additionally, correct calibration of the device remains challenging, but crucial to increase sensitivity and repeatability of measurements (Spencer and Morgan, 2020). Gökçe et al. recently compared IFC to flow cytometry (Gökçe et al., 2021). They highlighted that FCM has a higher specificity compared to IFC since the use of biomarkers allows a high-resolution differentiation between cells. In contrast, IFC is inherently a label-free technique, and its operation is fully electric. These two advantages provide the technique with considerable potential for automation, making it better suited for experiments that require continuous, real-time analysis (Spencer et al., 2020; Gökçe et al., 2021).

**Figure 9:**
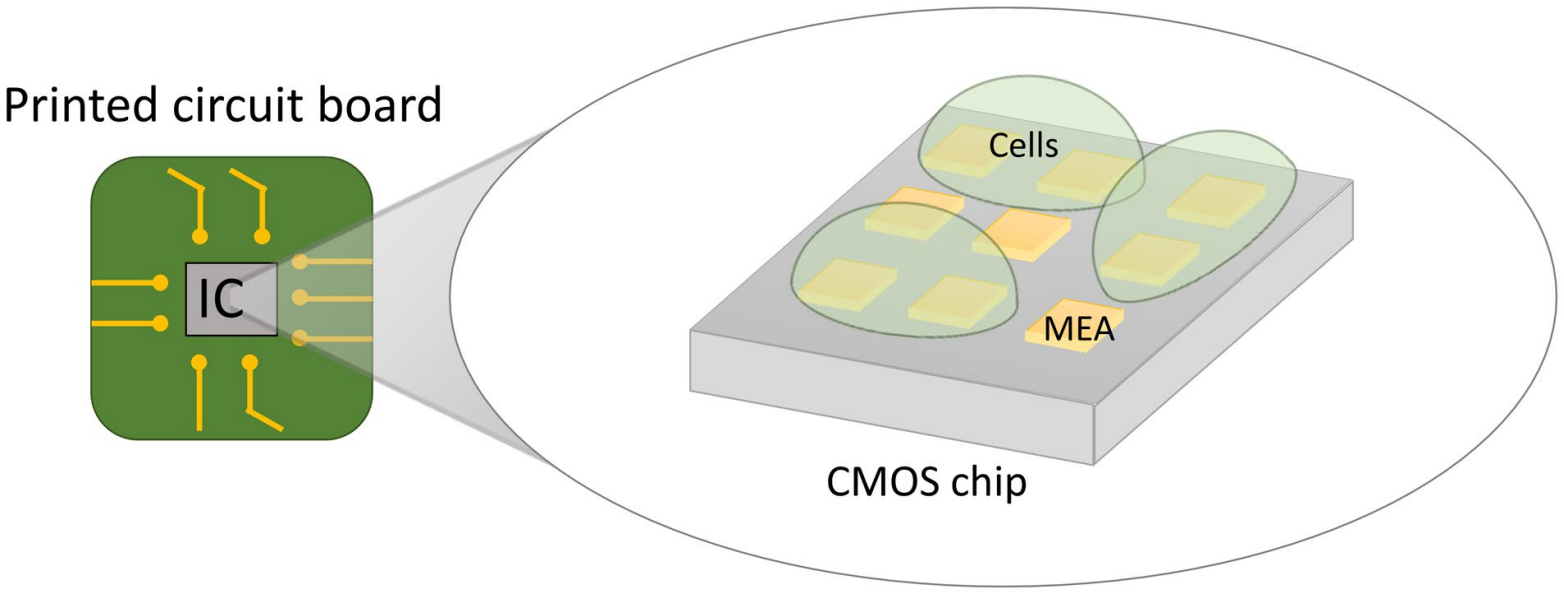
Illustration of the principle of a CMOS MEA device. A purpose-designed Complementary Metal-Oxide-Semiconductor (CMOS) integrated circuit (IC) is post-processed to encompass an array of microelectrodes (MEA) on its top surface. Each electrode is connected to the inner circuitry of the chip. The biological sample is placed on the electrode surface for fine-grained electrochemical characterization.

CMOS microelectrode arrays (MEA) are emerging as a novel technology for electrical single-cell analysis. Conventional electrochemical sensor systems consist of three parts: (i) the electrochemical sensor itself, comprised of working, counter and reference electrodes to interface with the sample under test, (ii) a measurement tool that generates and measures electrical signals (e.g., potentiostat), and (iii) cables connecting the electrochemical sensor to the measurement tool. In CMOS MEA’s, the required functionality of the measurement tool is implemented into a CMOS microchip, usually not bigger than 1 cm^2^. Tiny microelectrodes are post-processed on top of the microchip in a 2D array to serve as electrochemical sensors. The biological sample is placed on top of the microchip such that the electrode array contacts it (Figure 9). Each electrode in the array is connected to the inner circuitry of the chip by a tiny vertical connection called a “Vertical Interconnect Access” (via) (Birkholz et al., 2016). In conventional systems, there is a limit to how many electrodes can be connected to the measurement tool. Potentiostats rarely have more than ten channels since the cost of the system scales linearly with the amount of channels and having too many cables is simply impractical (Molderez et al., 2021). Furthermore, long cables introduce parasitic effects on the electrical signals such as parasitic capacitance and inductance which limit the sensitivity and speed of the measurement. In contrast, the microelectrodes on top of the CMOS microchip can be spaced closely together in a grid since they do not require cables to be connected. The electrodes are less than a millimeter away from the internal circuitry, which facilitates high-speed processing of the sensing signals. Additionally, the sensing circuitry can switch at high speed between the individually accessible electrodes to scan the array, thereby performing measurements almost in parallel. CMOS MEAs can therefore provide a high-resolution and real-time two-dimensional electrochemical image of the measured sample. The electrochemical measurement functionality integrated into the CMOS chip can differ enormously depending on the envisioned application. Examples of measurement functionality of CMOS MEAs include impedance spectroscopy, redox potential characterization, extracellular action potential recording, etc. Multiple functionalities are often integrated into the same chip providing a multifaceted analysis of the sample (Viswam et al., 2018; Abbott et al., 2022).

CMOS MEA devices intended for microbial applications have been demonstrated in the literature, but their functionality is generally restricted to cell detection and enumeration (Couniot et al., 2016; Gamo et al., 2017). The selectivity and specificity of the sensor can be drastically increased by functionalizing the electrodes with bio-recognition elements such as proteins, DNA strands or anti-bodies for the detection of pathogens (Manickam et al., 2010; Hsu et al., 2018; Furst and Francis, 2019). Other functionalities have been successfully explored such as electrochemical measurements on microbial biofilms by Kumashi et al. (2021). Their device was used to characterize the current generating capacity of exoelectrogenic bacteria, but the pixel size (100 μm x 100 μm) did not provide single-cell resolution. Ogawa et al. demonstrated an array of high frequency oscillators for monitoring the growth of *E. coli* (Ogawa et al., 2021). The small pixel area needed for single-cell bacteria measurements allows only limited space for in-pixel electronics necessary to amplify the small sensing currents (Niitsu et al., 2015). This makes it challenging to achieve an adequate signal-to-noise ratio on the sensing currents for measuring cell properties. Reports in the literature on CMOS MEA systems for cell analysis have primarily focused on eukaryotic cells rather than bacteria since the former are more convenient to measure due to their larger size. To apply the enormous potential of CMOS MEAs to microbial ecology, further advancements in circuit design are needed to implement complex measurement functionalities in bacteria-sized pixels. Such high-density microarrays provide researchers with the capability to augment the detection of bacteria at the single-cell level. Moreover, these microarrays have the opportunity to revolutionize our comprehension of microbial biofilms since they can provide high-resolution analysis of electrical properties, chemical processes, and growth dynamics. In the context of electrogenic bacteria, high-density microarrays present a unique platform for stimulating and characterizing their capacity for current generation.

The state of the art of CMOS MEA devices designed for eukaryotic cells can indicate potential future applications on bacteria. One application for CMOS MEAs is the characterization and stimulation of electrogenic cells (e.g., neurons and cardiomyocytes). These systems can locally stimulate cells by applying current spikes and recording intracellular and extracellular action potentials. Their electrodes can also be used to measure impedance and thereby generate a two-dimensional image of the cells on the electrodes. This image can be used to distinguish different cell layers of a brain slice (Viswam et al., 2018) or monitor cardiac cell contraction (Lopez et al., 2018). Another application for CMOS MEAs is monitoring the metabolic state of cancer cells in very high resolution. Recently, a device was reported for high-resolution measurements on a monolayer cell sheet of the extracellular redox potential, which allowed to differentiate between aerobic or anaerobic cell metabolism. These measurements were used to monitor the growth of a cell sheet in real-time and to study and compare the differences between normal and cancerous cells (Abbott et al., 2022). CMOS MEAs can also be used for comprehensive drug screening experiments. Chi et al. demonstrated their device by measuring the response of cardiac cells to the drug isoproterenol. They implemented multiple sensors in a single pixel to achieve the following four sensing modalities: voltage, impedance, optical and temperature measurements (Chi et al., 2015). CMOS MEAs with a submicron electrode pitch have been reported (Laborde et al., 2015; Widdershoven et al., 2018) but were not used for microbial applications. Although this technology is still in the early stages of development, commercial suppliers of CMOS MEAs exist, such as *MaxWell Biosystems* (^©^ 2020 MaxWell Biosystems AG, Switzerland), *Multichannel Systems* (© Multi Channel Systems MCS GmbH, Germany) and *3Brain* (^©^ 2022 3Brain AG, Switzerland). They offer high-density CMOS MEA’s for *in vitro* stimulation and recording of electrogenic cells.

## 7. Identifying new opportunities

Microbial cells can vary in their morphological, genetic, biochemical, physiological, or behavioral features, and recent advances in analytical techniques now enable microbiologists to uncover these differences with unprecedented precision. With methods capable of examining individual cells, researchers have gained important insights into microbial functions and their interactions with other microbes, higher organisms, and the environment.

As the field of microbiology keeps expanding, we summarize different techniques that can be used to derive information from samples and link them with current available bio-informatics tools in Table 1. Summarized techniques include flow cytometry, which can use unstained samples making use of auto fluorescence or scatter, or more advanced staining methods, such as BONCAT and FISH, as well as microscopy, Raman spectroscopy and the electrical techniques CMOS MEAs and impedance flow cytometry. FACS is not listed in the table as it can be seen as a derivative method of flow cytometry with similar data processing. The table discriminates between combinations that have been done and are reported in literature (checkmark), combinations that are practically impossible (cross), combinations worth to try (lightbulbs), and combinations that do not seem feasible at this time (question mark). It is important to note that binning approaches are not always directly applicable to the technique at hand. For example in image flow cytometry, deep learning approaches for image analysis can be used which do not make use of binning (Kelleher, 2019). This special case is indicated by the asterisk in the advanced binning approaches. For certain techniques, it may be possible to achieve certain information retrieval, but the technique itself may not be the most sensible to use for that purpose. For instance, one could perform spectral flow cytometry in combination with DNA staining to obtain information on nucleic acid content of cells. However, using conventional flow cytometry with a DNA staining would be sufficient for that purpose as well and be less complicated in terms of data analysis. Additionally, it is worth mentioning that within flow cytometry, scatter and fluorescence are often combined. Especially when considering fingerprinting approaches (to obtain within- and between-diversity), it is theoretically possible to use only fluorescence or scatter. However, this would lead to a loss of information and discriminative power.

**Table 1:** Overview of optical and electrical single-cell technologies for analysis of microorganisms and their data processing methods.

Bio-informatics		Cell counts	Manual gating	Low nucleic acid (LNA)/High nucleic acid (HNA)	Real-time	Within-diversity		Between-diversity	
						Standard binning	Advanced binning	Standard binning	Advanced binning
Techniques	Microscopy	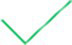 Schlundt et al. (2020)	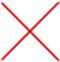	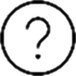 *	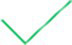 Ellison et al. (2019)	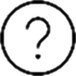 *	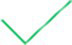 * Dhindsa et al. (2020)	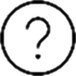 *	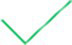 * Wimmer et al. (2023), Dhindsa et al. (2020)
	FCM – Auto fluorescence	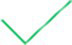 Paau et al. (1978), Patel et al. (2019), Ning et al. (2021)	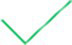 Paau et al. (1978), Patel et al. (2019), Ning et al. (2021)	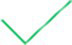 Paau et al. (1978), Patel et al. (2019), Ning et al. (2021)	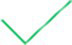 Thyssen et al. (2014), Paau et al. (1978), Patel et al. (2019), Ning et al. (2021)	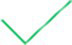 Patel et al. (2019), Ning et al. (2021)	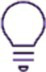	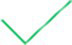 Patel et al. (2019), Ning et al. (2021)	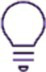
	FCM – Scatter	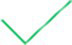 Ross (2021)	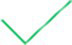 Ross (2021)	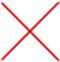	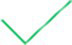 Thyssen et al. (2014)	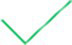 Props et al. (2016)	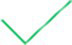 Rubbens et al. (2021), Futamura et al. (2015)	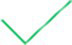 De Roy et al. (2012), Props et al. (2016), Buysschaert et al. (2018)	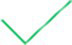 Rubbens et al. (2021), Futamura et al. (2015)
	FCM – DNA staining	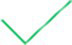 Brown et al. (2019), Ross (2021), Wang et al. (2009)	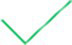 Props et al. (2016), Brown et al. (2019), Ross (2021), Wang et al. (2009)	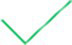 Wang et al. (2009), Besmer and Hammes (2016)	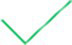 Sadler et al. (2020), Haberkorn et al. (2021), Buysschaert et al. (2018), Van Nevel et al. (2017)	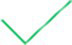 Props et al. (2016)	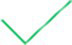 Rubbens et al. (2021), Futamura et al. (2015)	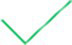 De Roy et al. (2012), Props et al. (2016), Buysschaert et al. (2018)	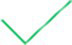 Rubbens et al. (2021), Futamura et al. (2015)
	FCM – Intact-damaged staining	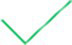 Van Nevel et al. (2017)	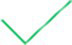 Van Nevel et al. (2017)	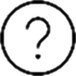	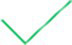 Freire et al. (2015), Buzatu et al. (2014)	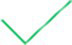 Van Nevel et al. (2017)	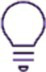	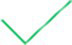 De Roy et al. (2012), Nevel et al. (2017)	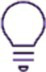 (manuscript in preparation)
	FCM – FlowFISH	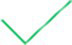 Heeren and Julian (2021), Rigottier-Gois et al. (2003)	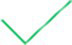 Heeren and Julian (2021), Rigottier-Gois et al. (2003)	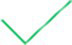 (target = RNA) Heeren and Julian (2021), Rigottier-Gois et al. (2003)	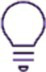	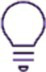 (manuscript in preparation)	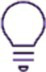	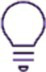 (manuscript in preparation)	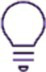 (manuscript in preparation)
	FCM – Activity staining	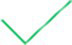 BONCAT: Lindivat et al. (2020, 2021)	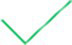 BONCAT: Lindivat et al. (2020, 2021)	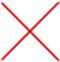	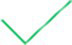 BONCAT: Lindivat et al. (2020)	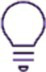	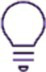	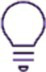	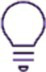
	Raman	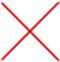	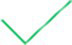 García-Timermans et al. (2020), Wang et al. (2020)	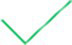 García-Timermans et al. (2020), Wang et al. (2020)	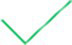 García-Timermans et al. (2020), Wang et al. (2020)	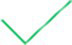 García-Timermans et al. (2020), Wang et al. (2020)	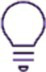	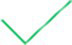 García-Timermans et al. (2020), Wang et al. (2020)	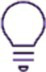
	IFC	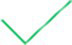 Haandbæk et al. (2014)	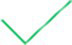 Clausen et al. (2018), Bertelsen et al. (2020)	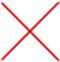	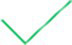 Spencer et al. (2020)	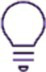	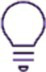	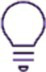	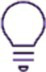
	CMOS MEA	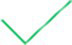 Couniot et al. (2016)	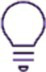 / 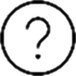	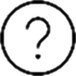	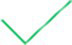 Kumashi et al. (2021)	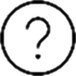 *	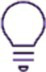 *	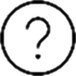 *	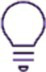 *
	Image FCM	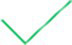 Buzatu et al. (2014), Pan and Kaatz (2012), Wnuk and Lewinska (2021)	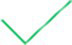 Buzatu et al. (2014), Pan and Kaatz (2012), Wnuk and Lewinska (2021)	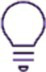 / 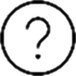	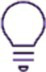	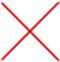	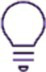 *	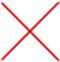	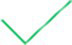 * Haridas et al. (2017), Luo et al. (2021)
	Spectral FCM	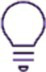	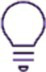	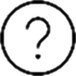	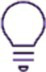	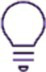	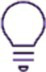	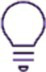	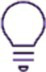

Checkmarks indicate specific combinations have been done before, crosses indicate physically impossible processes, lightbulbs indicate possibilities that have not been tried before, and question marks indicate that the combination may be possible but do not seem feasible at this time. Asterisk indicate that the technique does not use binning approaches for data analysis, but rather another form of advanced data analysis (e.g., deep learning).

As can be derived from Table 1, there is still a lot of unexplored possibilities for microbial analysis. For some techniques, like CMOS MEA, the possibilities will expand when technological advancements, in this case, smaller scale, will make them more suitable for microbial cells. Technologies such as spectral flow cytometry will be more important when complex combinations of color panels are used, for example when using antibodies for bacterial detection (Clarke and Pinder, 1998; Moor et al., 2016). At the same time, the availability of staining procedures for microbial cells is expanding, and therefore also are the applications of microbial flow cytometry. With the current available flow cytometric technology, there are still many possibilities to get more information out of data. For example advanced binning approaches will lead to better predictive capabilities of models, and these models could even develop into diagnostic tools. Additionally, integrative approaches to data analysis can be explored, combining different types of data on a sample level to increase predictive capabilities. This could be done by combining multiple fingerprints of a single sample, for example the cytometric fingerprint, the genotypic fingerprint and the physicochemical fingerprint.

Real-time applications on a single-cell level are becoming more important for proper control of microbial systems. To this end, the discussed technologies show excellent opportunities. This immediately leads to the question how real-time is real-time? For example, when using flow cytometry there may be a need to stain cells before analysis. This results in delays in time before the actual analysis on the machine. Taking into account that for example, *E. coli* can divide every 20 min in optimal laboratory conditions (Gibson et al., 2018), the posed research question can be influenced by this measurement delay. As mentioned earlier, fixatives could be a means to preserve the state of your culture when longer sample preparation is necessary. Nevertheless, fixation can influence the sample too. If certain processes are to be assessed while perturbations are introduced, one could opt to stain the samples before the perturbations are applied. However, there is a need for the discretization of time which adds to the analysis time. It is important to mention that this may become less important given that the analysis speed of flow cytometers is becoming faster [e.g., the Invitrogen Attune NxT flow cytometer can acquire up to 35,000 events/s (Invitrogen, 2021)]. Moreover, when talking about online monitoring of microbial systems, the time to analyze the data must be considered as well. For example, in drinking water quality monitoring, the so-called time-to-results can range from 10 min to 2.5 h depending on the online microbial monitoring technique used (Favere et al., 2021). Even in an automated data analysis setting, there is still time used for actual computation because these datasets can be big and fingerprinting calculations can take considerable computational power. On the other hand, computational power keeps increasing as computer technology is becoming better every day.

Label-free electrical techniques can open new opportunities in this regard. A cell label does not only increase the preparation time, but can also alter or inhibit certain cell functions. Monitoring the real-time response of a cell population therefore requires the use of a label-free technique to obtain reliable results. The electrical techniques described above can provide an important platform to characterize such dynamic changes in a population. More specifically, they can be used to increase our understanding of how microbial populations respond to different environments or drugs (Gökçe et al., 2021). IFC systems provide an excellent platform for such experiments on suspended cells (Spencer et al., 2020). CMOS MEAs on the other hand present the opportunity to characterize, with high-resolution, the real-time response of adherent cells such as biofilms.

These electrical technologies also have the potential to be used as portable devices that will allow analysis of microbial systems *in situ*. Similar to real-time monitoring, *in situ* analysis reduces the possibility of changes occurring within samples, as these do not need to be transported to be analyzed. Aforementioned electrical techniques especially seem suitable for this purpose, because miniaturization of electronics is already advanced and they do not make use of consumables and sample preparation to the same extend that the optical techniques do. However, efforts are being made to miniaturize Raman spectroscopy and flow cytometry as well, with their main advantage being that their sensitivity is higher compared to the electrical techniques (Lapsley et al., 2013; Persichetti et al., 2017; Shrirao et al., 2018; Hao et al., 2020; Gökçe et al., 2021; Jin et al., 2021; Li et al., 2023; Park et al., 2023). Moreover, flow cytometers and Raman spectroscopes are becoming cheaper (Lam, 2004; Shapiro, 2004; Picot et al., 2012; Emmanuel et al., 2021), paving the way for cheap and portable microbial analysis.

Cheap and fast microbial analysis of discussed optical and electrical techniques is the most prominent advantage over current sequencing technologies. Moreover, it is important to note that the type of information gained from optical and electrical techniques is different in nature. These techniques provide phenotypic and/or metabolic information, as opposed to genotypic or translational information from sequencing techniques. This means that changes in microbial communities can be detected earlier on (Sabbe et al., 2023). For example, the response sensitivity of *E. coli* of chemoreceptors Tar and Tsr can be modulated posttranslational and depends on environmental factors (Kamino et al., 2020). While it could be observed by single-cell FRET microscopy, sequencing technologies will not be able to pick up the change. Additionally, as indicated before, most of the optical and electrical techniques offer (semi-) quantitative information, while this cannot be achieved by conventional sequencing methods (Knight et al., 2018). Last, the optical and electrical techniques result in information collected on a cell-per-cell basis, whereas sequencing provides information only on the bulk of the community. This leads to higher resolution when trying to understand the functioning of microbial communities. However, new developments in single-cell sequencing for microbial samples will also lead to information with resolution at a cell-per-cell level (Lloréns-Rico et al., 2022).

## 8. Conclusion

Optical and electrical single-cell technologies are increasingly applied for the study of microbial ecology. In this review, we highlighted the strengths of microscopy, flow cytometry and FACS, Raman spectroscopy, impedance flow cytometry and CMOS MEA in order to assist the study, control and engineering of microbial populations. We demonstrated that information obtained through these techniques holds great value and can be used for addressing different research questions. Simultaneously, the limitations and challenges of each technique are recognized and insight in optimization and future developments is provided. We identified interesting and novel opportunities for applications, both on the level of the technique and in its bio-informatics processing. Summarized, we provided an overview to guide researchers towards the correct method for their microbial ecosystem applications and motivate scientists to expand knowledge on un(der)explored possibilities.

## Author contributions

FM, VM, RE, CG-T, JL, and HK: drafting manuscript. FM, VM, YG, HK, and NB: conceptualization. FM, VM, RE, CG-T, JL, YG, and HK: figures. FM and VM: table. FM, VM, RE, CG-T, JL, YG, IT, FT, MK, HK, and NB: review and editing manuscript. All authors contributed to the article and approved the submitted version.

## Funding

FM is funded by Research Foundation - Flanders (FWO, Belgium) (grant number 3G0B2719). VM is funded by the Flemish Agency for Innovation & Entrepreneurship (VLAIO, Belgium) and B4Plastics (Dilsen-Stokkem, Belgium) via a Baekeland Ph.D. fellowship (grant number HBC.2019.2622). RE is funded by Research Foundation - Flanders (FWO, Belgium) (grant number G020119N). CG-T is funded by Research Foundation - Flanders (FWO, Belgium) (grant number S006221N). JL is funded by Special Research Fund (Ghent University, Belgium) (grant number BOF.STG.2021.0041.01). HK is funded by Research Foundation - Flanders (FWO, Belgium) (grant number 3G020119).

## Conflict of interest

The authors declare that the research was conducted in the absence of any commercial or financial relationships that could be construed as a potential conflict of interest.

## Publisher’s note

All claims expressed in this article are solely those of the authors and do not necessarily represent those of their affiliated organizations, or those of the publisher, the editors and the reviewers. Any product that may be evaluated in this article, or claim that may be made by its manufacturer, is not guaranteed or endorsed by the publisher.
